# Epigenetic age acceleration correlates with BMI in young adults

**DOI:** 10.18632/aging.204492

**Published:** 2023-01-18

**Authors:** Christy Anne Foster, Malcolm Barker-Kamps, Marlon Goering, Amit Patki, Hemant K. Tiwari, Sylvie Mrug

**Affiliations:** 1 Department of Pediatrics, University of Alabama at Birmingham, Birmingham, AL 35233, USA

**Keywords:** obesity, epigenetic aging, young adult, DNA methylation, epigenetic acceleration

## Abstract

Introduction: Obesity increases the risk of Type 2 diabetes, cardiovascular disease, several types of cancer, and other age-related disorders. Among older adults, obesity is also related to epigenetic age, typically measured with DNA methylation (DNAm). Because less is known about obesity and epigenetic aging earlier in the lifespan, this study examined the relationship between obesity and DNAm in young adulthood and whether these relationships vary by sex.

Methods: A cross-sectional community sample of 290 healthy young adults (mean age 27.39 years, 60% female; 80% African American, 18% White) had their BMI and waist circumference measured. Four epigenetic age estimators were derived from salivary DNA: Hannum DNAm, Horvath DNAm, Phenoage DNAm, and GrimAge DNAm. Sociodemographic covariates included age, sex, race, parental education, and income-to-needs ratio.

Results: After adjusting for covariates, higher BMI and waist were associated with higher DNAm PhenoAge in both sexes, with a stronger effect on BMI in males (β = 0.35, *p* < .001) compared to females (β = 0.13, *p* = .002). Higher waist, but not BMI, was associated with higher Horvath DNA methylation age. Both BMI and waist circumference were associated with higher Hannum DNAm age in men but not in women. Neither BMI nor waist circumference were related to GrimAge.

Discussion: This study extends prior research by linking obesity with accelerated epigenetic aging in young adulthood, replicating the associations across two measures of obesity and four indices of salivary epigenetic aging. The results add to evidence that higher BMI accelerates aging early in the lifespan.

## INTRODUCTION

Between 2017–2018, approximately 45% of adults in the United States were classified as obese, placing them at increased risk of functional decline and morbidity [1]. Obesity increases the risk of various comorbidities, including Type 2 diabetes, cardiovascular disease, and several types of cancer including liver cancer, breast cancer and esophageal cancer [2]. Obesity also increases the risk for many age-related disorders [3]. With the development of the field of epigenetics, epigenetic age acceleration has also been linked to obesity [4].

DNA methylation-based biomarkers satisfy the criteria of a molecular biomarker of aging which can be applied to different tissues across the spectrum of all ages [5]. The methylation states of millions of CpG dinucleotides in the human genome were shown to change with age [6–9]. Accelerated biological aging, which may be measured through a multiple biomarker approach, is likely to result from multiple physiological and pathological changes, including obesity, during the life-course, and therefore may represent an overarching mechanism linking obesity and health. For example, obesity has been linked with methylation changes in genes involved in lipid metabolism (*ABCG1, SREBF1, and NOD2*), which may explain the comorbidities noted in obesity [10].

Epigenetic age estimators are sets of CpGs that are coupled with a mathematical algorithm to estimate the age in years of a DNA source. For example, Hannum et al. derived a highly accurate age estimator based on 71 CpGs from whole blood DNA, known as the Hannum DNAm [11]. Another methylation aging index, Horvath’s DNAm, was trained and validated for predicting age using 8,000 publicly available microarray samples from over 30 different tissue and cell types collected from children and adults [5]. Horvath epigenetic aging was also positively correlated with total cholesterol, HDL, and triglycerides and inversely correlated with fasting HDL [12].

Another approach to studying methylation aging involves the replacement of chronological age with a surrogate measure of biological age (‘phenotypic age’) that differentiates morbidity and mortality risk among individuals of the same age, as exemplified by the phenotypic age estimators PhenoAge and GrimAge built by Levine et al. and Lu et al., respectively [13, 14]. Both DNAm PhenoAge and GrimAge outperformed the first generation of DNAm age estimators in predicting mortality, health span and cardiovascular disease, as well as various measures of multimorbidities including cognitive impairment, cancers and Alzheimer’s syndrome [13, 14].

Multiple studies have examined associations between the aforementioned DNAm age indices and obesity. For example, using the Horvath clock, Horvath et al. linked BMI with greater epigenetic age acceleration in 1,215 adults aged 37–77 [15]. Nevalainen et al. showed that accelerated epigenetic age (using the Horvath clock) is correlated with higher BMI in midlife (ages 40–49) and with increased BMI from ages 15–24 to midlife, but not with BMI at ages 15–24 and age 90 [16]. However, BMI was associated with higher Horvath clock epigenetic aging from salivary DNA in 232 African American mothers (mean age 31 years) [17]. Moreover, Quach et al. found associations between BMI and both Hannum and Horvath accelerated epigenetic aging indices in 2,725 postmenopausal women (ages 50–82) from the Women’s Health Initiative, although these results were not replicated among 402 males and females from the Italian InCHIANTI cohort (ages 30–100) [18]. Finally, a recent study of 273 maltreated children (ages 8–14) suggests that the association between BMI and epigenetic aging may emerge as early as in childhood [19]. Despite these few studies with younger groups, the majority of research on obesity and epigenetic aging has focused on middle-aged and older adults. Investigating DNAm aging earlier in the lifespan, before the onset of chronic diseases, is critical to guide interventions that could potentially improve later health outcomes. Moreover, few studies have included replication across measures of obesity and epigenetic aging to examine the robustness or specificity of these effects. Finally, little is known about sex differences in the links between obesity and epigenetic aging, despite evidence of substantial sex dimorphism in both physiological and epigenetic aging [20].

This study addressed gaps in the literature by using a racially diverse community sample of healthy young adults to examine the relationship between two measures of obesity (BMI and waist circumference) and four most widely used indices of epigenetic aging – Hannum, Horvath, PhenoAge DNAm, and GrimAge DNAm in young adulthood, prior to onset of chronic disease. In addition, this study tested sex differences in the associations between BMI and DNAm aging. It was hypothesized that higher BMI and waist circumference would be associated with accelerated DNAm aging in young adults, with stronger associations in males who generally show faster epigenetic aging [21].

## RESULTS

### Preliminary analyses

Among the 290 participants included in the analyses, 268 (92%) had complete data on all variables and less than 1% of data points were missing. Individuals with missing data did not differ from participants with complete data on any variables included in the analyses. Descriptive statistics and bivariate correlations among the variables included in the analyses are presented in Table 1. Bivariate correlations including epigenetic age indices were adjusted with chronological age. Higher PhenoAge was correlated with higher Hannum and higher Horvath DNAm age. Horvath DNAm was associated with higher waist circumference and PhenoAge was correlated with higher BMI. Horvath DNAm age was associated with higher chronological age at Wave 4. Females had higher PhenoAge and lower GrimAge scores than males, while African Americans had lower Hannum DNAm and higher GrimAge age scores than Whites. Females had higher BMI than males.

**Table 1:** Descriptive statistics and bivariate correlations among all variables.

	**M (SD)**	**1**	**2**	**3**	**4**	**5**	**6**	**7**	**8**	**9**	**10**	**11**	**12**	**13**	**14**	**15**	**16**
1. Age	27.39 (1.19)																
2. BMI	31.59 (11.44)	−0.06															
3. Waist	98.04 (19.88)	−0.02	0.82^*^														
4. Parental education	4.30 (1.70)	−0.26^*^	0.00	−0.07													
5. Income-to-needs ratio	6.55 (3.82)	−0.21^*^	0.04	−0.03	0.26^*^												
6. African American	80%	0.21^*^	0.06	−0.01	−0.20^*^	−0.25^*^											
7. Horvath DNAm age	35.28 (4.36)	0.29^*^	0.09	0.18^*^	−0.06	−0.06	−0.11										
8. Hannum DNAm age	40.35 (3.96)	−0.04	−0.01	0.01	0.03	0.04	−0.15^*^	0.11									
9. PhenoAge	29.70 (6.13)	0.07	0.16^*^	0.12^*^	0.02	−0.12^*^	−0.01	0.16^*^	0.27^*^								
10. GrimAge	50.13 (5.75)	0.37^*^	−0.06	−0.09	−0.13^*^	−0.15^*^	0.16^*^	0.16^*^	−0.08	0.31							
11. Smoking	1.05 (0.83)	0.06	−0.03	0.00	−0.10	−0.03	−0.16^*^	0.04	−0.04	−0.03	0.17						
12. Female	60%	−0.02	0.16^*^	0.09	−0.11	−0.13^*^	0.15^*^	0.07	0.04	0.18^*^	−0.13^*^	−0.24^*^					
13. CD8T	0.01 (0.03)	0.03	−0.04	−0.07	0.03	−0.10	0.03	0.03	0.00	0.33^*^	0.68^*^	−0.10	0.03				
14. CD4T	0.05 (0.05)	−0.21^*^	−0.10	−0.15^*^	0.11	0.06	0.00	−0.20^*^	0.48^*^	0.41^*^	0.27^*^	−0.06	−0.10	0.35^*^			
15. Bcell	0.07 (0.04)	−0.18^*^	−0.10	−0.14^*^	0.14^*^	0.00	0.02	−0.16^*^	0.48^*^	0.41^*^	0.46^*^	−0.13^*^	−0.06	0.68^*^	0.86^*^		
16. Monocyte	0.07 (0.03)	−0.18^*^	−0.03	−0.08	0.12^*^	0.03	0.18^*^	−0.19^*^	0.45^*^	0.40^*^	0.17^*^	−0.14^*^	0.07	0.21^*^	0.85^*^	0.70^*^	
17. Gran	0.82 (0.12)	0.15^*^	0.09	0.14^*^	−0.12	0.00	-0.07	0.14^*^	−0.38^*^	−0.10	−0.49^*^	0.13^*^	0.01	−0.67^*^	−0.91^*^	−0.96^*^	−0.81^*^

All correlations involving epigenetic clock variables (Horvath, Hannum, PhenoAge, and GrimAge) are adjusted for participants’ age. ^*^p < .05 or lower.

### Main analyses

#### BMI and epigenetic aging

**Figure 1:**
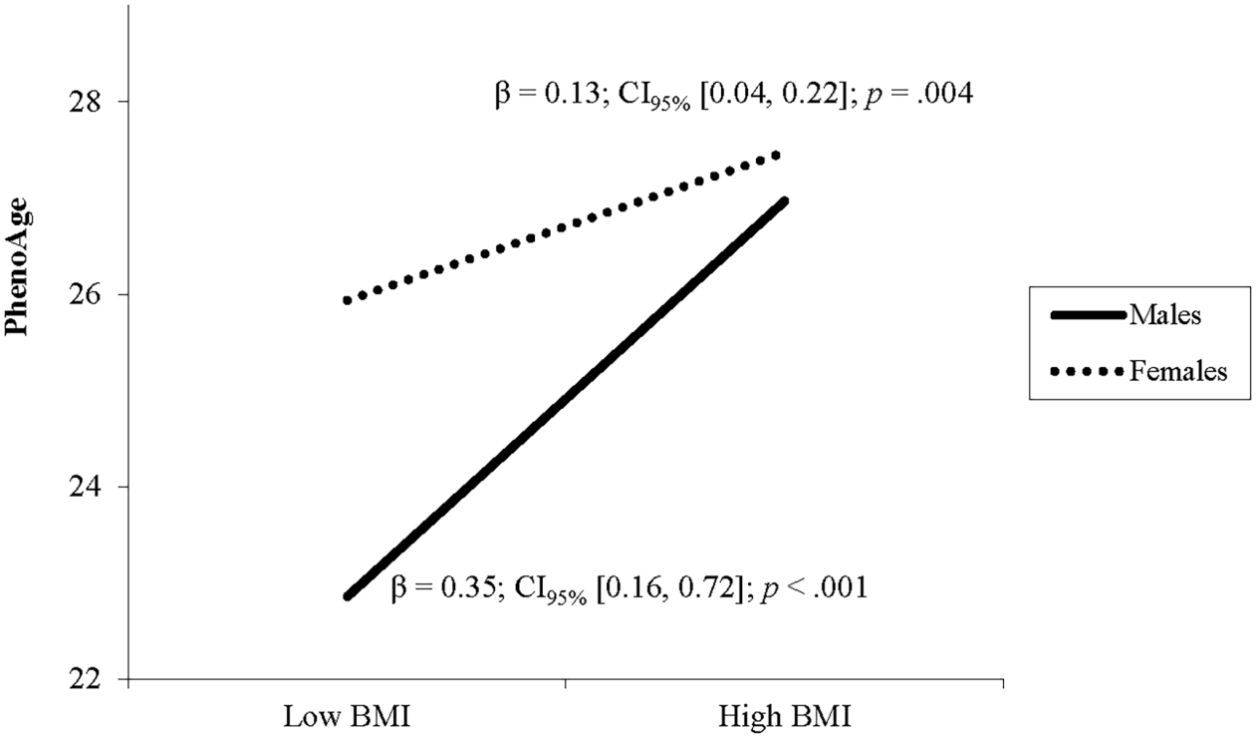
**The relationship between BMI and PhenoAge varies by sex.** Note: Low and high BMI were defined as 1 SD below and above the mean.

After adjusting for covariates, higher BMI was associated with greater DNAm PhenoAge (Table 2). A significant interaction of BMI and sex indicated that the relationship between BMI and DNAm PhenoAge differed between males and females. Follow up simple slope analyses demonstrated that the association between BMI and DNAm PhenoAge was significant in both sexes but was stronger in males (β = 0.35, *p* < .001) compared to females (β = 0.13, *p* = .002; see Figure 1).

**Table 2:** Hierarchical regression models predicting DNA methylation aging from BMI and covariates.

**Variable**	**GrimAge β [CI_95%_]**	**PhenoAge β [CI_95%_]**	**Horvath DNAm age β [CI_95%_]**	**Hannum DNAm age β [CI_95%_]**
Step 1				
BMI	0.02 [−0.06, 0.07]	0.19^***^ [0.11, 0.27]	0.08 [−0.05, 0.21]	0.02 [−0.06, 0.10]
Age	0.27^***^ [0.14, 0.38]	0.15^**^ [0.05, 0.25]	0.24^***^ [0.13, 0.34]	0.17^**^ [0.07, 0.26]
Female	−0.22^***^ [−0.313, −0.121]	0.15^**^ [0.05, 0.26]	−0.05 [−0.16, 0.07]	0.13^*^ [0.01, 0.25]
African American	0.13^**^ [0.041, 0.21]	−0.13^*^ [−0.23, -0.03]	−0.13^*^ [−0.24, −0.02]	−0.24^***^ [−0.34, −0.13]
Income-to-needs ratio	−0.10^*^ [−0.18, -0.02]	−0.13^*^ [−0.23, -.02]	−0.07 [−0.18, 0.03]	0.00 [−0.09, .10]
Parental education	−0.01 [−0.08, 0.05]	0.02 [−0.09, 0.13]	−0.01 [−0.11, 0.10]	−0.05 [−0.15, 0.05]
Smoking	0.20^***^ [0.10, 0.30]	0.04 [−0.06, 0.14]	−0.02 [−0.13, 0.10]	−0.02 [−0.12, 0.08]
CD8T	−0.69^***^ [−1.04, −0.32]	0.05 [−0.43, 0.54]	−0.57 [−1.23, 0.10]	0.21 [−0.46, 0.88]
CD4T	−1.53^***^ [−2.02, −1.00]	0.16 [−0.37, 0.68]	−0.87^*^ [−1.57, −0.18]	0.80 [−0.52, 2.11]
Monocytes	−1.17^***^ [−1.55, −0.76]	−0.05 [−0.45, 0.36]	−0.70^**^ [−1.20, −0.20]	0.70 [−0.09, 1.49]
B cells	−1.62^***^ [−2.09, −1.13]	−0.40 [−0.93, 0.12]	−1.08^***^ [−1.73, −0.42]	1.07^*^ [0.19, 1.94]
Granular cells	−4.80^***^ [−6.07, −3.45]	−0.74 [−2.19, 0.70]	−2.64^**^ [−4.51, −0.76]	2.04 [−0.79, 4.87]
Step 2				
BMI X Female	0.06 [−0.15, 0.28]	−0.36^*^ [−0.69, −0.03]	−0.35 [−.77, .07]	−0.36^**^ [−0.64, −0.09]

Significant coefficients are bolded. Standardized coefficients and confidence intervals are shown. ^*^p < .05, ^**^p <.01, ^***^p <.001.

**Figure 2:**
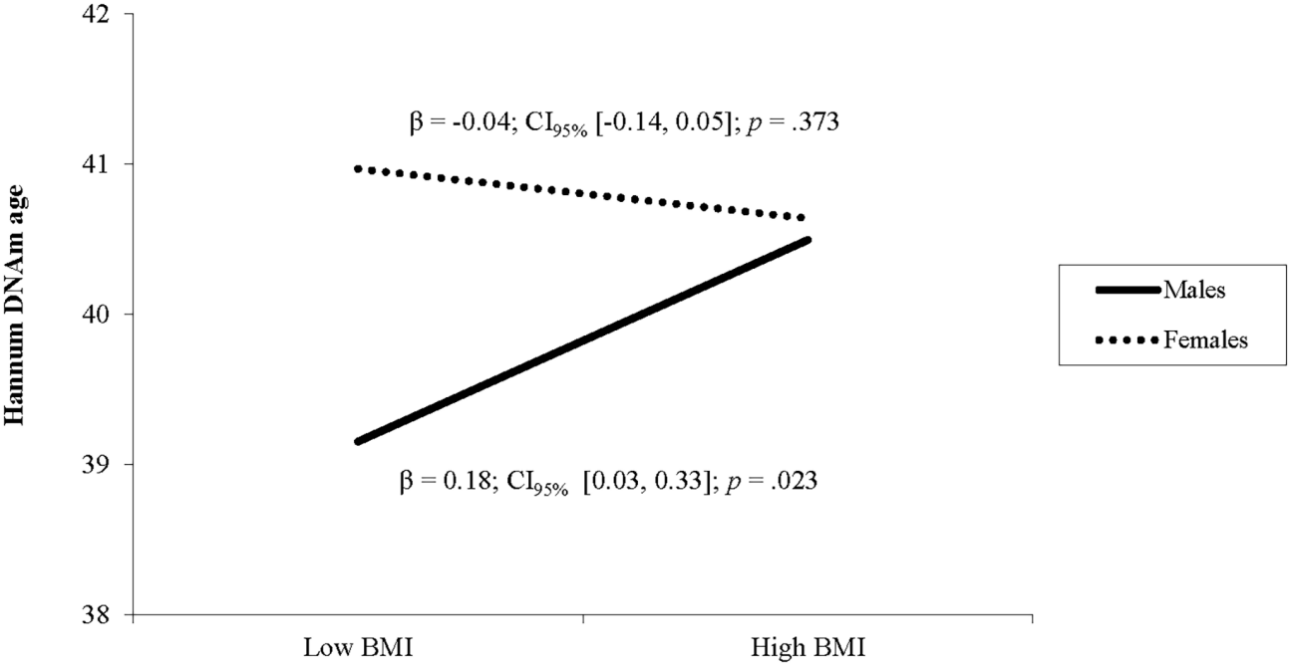
**The relationship between BMI and Hannum DNAm varies by sex.** Note: Low and high BMI were defined as 1 SD below and above the mean.

In the second model, BMI was not related to Horvath DNA methylation age and there was no interaction between BMI and biological sex (see Table 2). In the third model, BMI was not a unique predictor of Hannum DNAm age, but there was a significant interaction between BMI and sex (see Table 2). Follow up simple slope analyses showed that BMI was associated with higher Hannum DNAm age in males (β = 0.18, *p* = .023) but not in females (β = −0.04, *p* = .373; see Figure 2). In the fourth model, BMI was not related to GrimAge and there was no interaction between BMI and sex (see Table 2).

Among the covariates, older chronological age was associated with higher epigenetic aging across all four indices. Women had higher epigenetic aging on PhenoAge and Hannum DNAm but lower on GrimAge. A higher income-to-needs ratio was related to lower PhenoAge and GrimAge. African Americans had lower Phenoage, Horvath DNAm age and Hannum DNAma higher scores but higher GrimAge scores. Horvath DNA methylation age was also uniquely associated with lower proportions of CD4T cells, monocytes, B cells, and granular cells, whereas Hannum DNAm age was related to the greater proportion of B-cells.

Sensitivity analyses using BMI scores standardized within each sex replicated the relationship between BMI and higher PhenoAge (β = 0.20, *p* < .001), but the BMI by sex interaction was no longer significant (β = −0.12, *p* = .130). The sensitivity analyses also replicated no unique association between BMI and Horvath DNAm age (β = −0.14, *p* = .207), as well as no sex differences in this association (β = −0.14, *p* = .207). In addition, the results replicated the BMI by sex interaction for Hannum DNAm age (β = −0.15, *p* = .015), with BMI being associated with higher Hannum DNAm age in males (β = 0.15, *p* = .019) but not in females (β = −0.05, *p* = .376). The sensitivity analyses showed no interaction of BMI and sex for GrimAge (β = 0.05, *p* = .760). Finally, sensitivity analyses showed no interaction effects of BMI with race for any of the four epigenetic clocks.

#### Waist circumference and epigenetic aging

**Figure 3:**
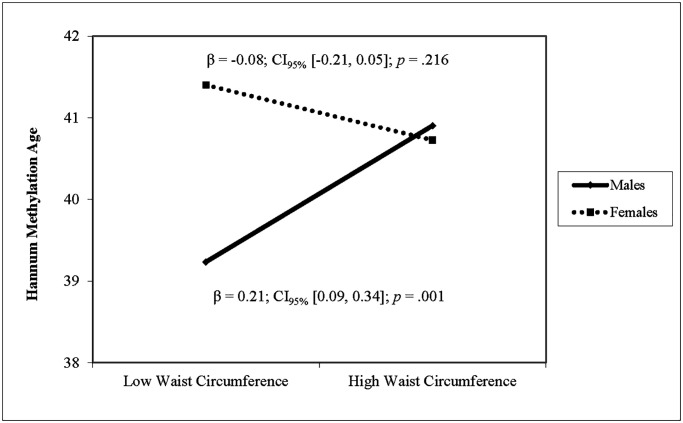
**The relationship between waist circumference and Hannum DNAm varies by sex.** Note: Low and high waist circumference were defined as 1 SD below and above the mean.

After adjusting for covariates, higher waist circumference was associated with higher DNAm PhenoAge and higher Horvath DNAm age (see Table 3). For Hannum DNAm age, there was a significant interaction of waist circumference with sex. Simple slope analyses revealed that larger waist circumference was associated with higher Hannum methylation age in males (β = 0.21, *p* < .001) but not in females (β = −0.08, *p* = .216) (see Figure 3). Waist circumference was not associated with GrimAge and there was no interaction of waist circumference and sex. Sensitivity analyses using waist circumference standardized within sex yielded identical results. Sensitivity analyses showed no interaction effects of waist circumference with race on any of the four epigenetic clocks.

**Table 3:** Hierarchical regression models predicting DNA methylation aging from waist circumference and covariates.

**Variable**	**GrimAge β [CI_95%_]**	**PhenoAge β [CI_95%_]**	**Horvath DNAm age β [CI_95%_]**	**Hannum DNAm age β [CI_95%_]**
Step 1				
Waist	0.01 [−0.06, 0.07]	0.17^***^ [0.08, 0.26]	0.18^**^ [0.08, 0.28]	0.04 [−0.05, 0.14]
Age	0.26^***^ [0.14, 0.38]	0.14^**^ [0.04, 0.25]	0.24^***^ [0.13, 0.34]	0.17^**^ [0.07, 0.26]
Female	−0.22^***^ [−0.31, −0.12]	0.17^**^ [0.07, 0.27]	−0.05 [−0.16, 0.06]	0.13^*^ [0.01, 0.25]
African American	0.13^**^ [0.04, 0.21]	−0.11^*^ [−0.22, −0.01]	−0.12^*^ [−0.23, −0.01]	−0.24^***^ [−0.34, −0.13]
Income-to-needs ratio	−0.10^*^ [−0.18, −0.02]	−0.11^*^ [−0.22, −0.01]	−0.07 [−0.18, 0.04)	0.01 [−0.09, 0.10]
Parental education	−0.01 [−0.08, 0.05]	0.02 [−0.09, 0.14]	0.01 [−0.10, 0.11]	−0.05 [−0.15, 0.05]
Smoking	0.20^***^ [0.10, 0.30]	0.04 [−0.06, 0.14]	−0.02 [−0.13, 0.09]	−0.03 [−0.12, 0.07]
CD8T	−0.68^***^ [−1.04, −0.32]	0.07 [−0.42, 0.57]	−0.64 [−1.30, 0.03]	0.20 [−0.48, 0.87]
CD4T	−1.51^***^ [−2.02, −1.00]	0.20 [−0.34, 0.73]	−0.92^**^ [−1.59, −0.25]	0.79 [−0.53, 2.09]
Monocytes	−1.16^***^ [−1.55, −0.76]	−0.05 [−0.47, 0.37]	−0.79^**^ [−1.28, −0.30]	0.68 [−0.12, 1.47]
B cells	−1.61^***^ [−2.09, −1.13]	−0.43 [−0.97, 0.11]	−1.16^***^ [−1.81, −0.51]	1.05^*^ [0.17, 1.93]
Granular cells	−4.76^***^ [−6.07, −3.45]	−0.72 [−2.19, 0.75]	−2.89^**^ [−4.72, −1.07]	1.98 [−0.86, 4.82]
Step 2				
Waist X Female	0.02 [−0.04, 0.15]	−0.22 [−0.71, 0.27]	−0.33 [−0.84, 0.19]	−0.76^**^ [−1.22, −0.29]

Significant coefficients are bolded. Standardized coefficients and confidence intervals are shown. ^*^p < 0.05, ^**^p < 0.01, ^***^p < .001.

## DISCUSSION

The present study examined the relationships between BMI, waist circumference, sex, and four measures of epigenetic age acceleration in a racially diverse sample of healthy young adults. Using the PhenoAge biological clock, both BMI and waist circumference were associated with higher DNAm. Additionally, both BMI and waist circumference were related to higher Hannum DNAm in males, but not in females. Using the Horvath DNAm, waist circumference but not BMI was associated with DNAm aging in both sexes. However, neither BMI nor waist circumference were related to the GrimAge epigenetic clock, and none of the associations of BMI and waist circumference with the DNAm indicators varied by race. Finally, higher DNAm aging on one or more of the four indices was associated with older chronological age, lower socioeconomic status, female sex, and White race, as well as saliva cell composition. Together, these results suggest that higher BMI and waist circumference are associated with higher epigenetic age in young adulthood. Because the analyses adjusted for chronological age, associations with higher epigenetic age indicate faster epigenetic aging [22]. Importantly, this study demonstrated associations between obesity and epigenetic aging using DNA from saliva, which involves a non-invasive sample collection compared to other tissues (e.g., blood) and thus can be more readily translated into clinical practice, highlighting the usefulness in young adults.

The present results extend findings of prior studies which demonstrated associations between higher BMI and accelerated epigenetic age in middle-aged and older adults utilizing the Hannum and Horvath clocks [4, 15]. Importantly, the relationship between obesity and epigenetic aging has been demonstrated across multiple tissues, from saliva cells in this study to liver tissue [15], brain and heart tissues [5], and visceral adipose tissue [4]. The convergence of these findings suggests that obesity contributes to accelerated aging across multiple body systems.

These findings may help explain how higher BMI or adiposity contribute to aging-related conditions such as diabetes and the shortening of telomeres [23, 24]. The role of BMI in overall lifespan has been extensively investigated. For example, both the length of time spent in a high BMI state and lifetime peak BMI have a positive relationship with all-cause mortality [25]. One mechanism through which obesity-related accelerated epigenetic aging may contribute to morbidity is through inflammation [12]. Age-related changes in inflammation are believed to contribute to increased risk of a myriad of comorbidities later in life, including diabetes, cardiovascular disease, and some cancers [26, 27].

Studies have found evidence that obesity is more robustly associated with accelerated epigenetic aging in males compared to females, at least for the Hannum clock. However, females in this study had more accelerated aging on the PhenoAge and Hannum DNAm clocks than males, in contrast to prior studies reporting that women have lower DNA-methylation age than men across the lifespan across the Horvath, Hannum, and PhenoAge clocks [21, 28]. Nevertheless, GrimAge showed lower DNA-methylation age in females, which is consistent with other findings [14, 29]. Among other covariates, higher chronological age, lower income-to-need ratio, and White race were associated with older epigenetic age, in line with prior studies [30, 31], with the exception of GrimAge linking White race with younger epigenetic age. The present findings further add to existing evidence that associations of risk factors with epigenetic aging differ by type of epigenetic aging index [32], consistent with the fact that these epigenetic clocks reflect different aspects of the aging process and aggregate different methylation sites [21]. Together, these findings underscore the importance of including multiple epigenetic clocks in the same study for a more comprehensive understanding of biological aging and related risk and protective factors.

Given that epigenetic aging predicts mortality and morbidity, the estimated epigenetic clocks can be used to identify at risk patients who might benefit from lifestyle changes or other therapeutic interventions. Interestingly, studies have begun to examine the effects of lifestyle interventions on epigenetic aging. For example, a weight-loss intervention in a small sample of obese older adults led to decreased DNAm age that was associated with improved gait speed and grip strength [33]. Additionally, following a Mediterranean-like diet for one year showed a trend toward slowing epigenetic age in some subgroups of participants [34].

The present study has several limitations. First, the sample size was smaller relative to some other studies of BMI and epigenetic aging [5, 15, 18], which may have limited statistical power to detect small effects. The limited sample size also precluded the examination of less widely used epigenetic clocks which would inflate Type I error. Likewise, the sample size did not allow a rigorous analysis of individual CpG sites with correction for multiple comparisons. Future, larger studies should replicate the present results and extend them by examining other epigenetic clocks and individual CpG sites with appropriate corrections for multiple testing. Second, the original sample was locally representative but experienced some differential attrition over time, with females, African Americans, and individuals from higher educational backgrounds being more likely to be retained over time. Thus, the findings may be less generalizable to males, non-African Americans, and individuals from lower socio-economic backgrounds. Additionally, the epigenetic clocks have been tested primarily in White populations, so they may be less relevant to African American individuals who comprised the majority of this sample. Although this issue was partly addressed in the sensitivity analyses testing race as a moderator, some racial bias in the results may remain. Future studies should develop and validate epigenetic clocks using more diverse samples. Next, this study used salivary DNA, so replication using DNA extracted from other tissues will be important in future work. For instance, GrimAge was trained in blood samples [14], which may explain why it yielded different results than the other epigenetic clocks in this study. Finally, the cross-sectional design did not allow testing of directional effects between BMI and epigenetic aging over time. Looking into the literature, multiple studies have looked at the directionality of effects between CpGs from EWAS and obesity with Mendelian randomization. There were 3 categories: BMI genes, methylation score-based studies, and studies where BMI was noting the primary outcome. The Mendelian randomization supports that maternal glycemia is part of a causal pathway influences offspring leptin epigenetic regulation. Ideally to understand the methylation aging, the model should be extended to include the outcomes in tissues impacted with obesity. There was no concordance of *SOCS3*, *JAK2*, *ATP4A*, and *ABCG1* CpGs and methylation aging. Future studies should use longitudinal designs to help elucidate directional relationships between obesity and epigenetic aging across the lifespan. None of the CpGs used in calculating methylation age were part of CpG known to have a causal effect on BMI used in Mendelian Randomization studies of BMI. Potential further modeling to include outcomes with other tissues may be helpful to further understand the outcomes.

In conclusion, this study extends prior research by demonstrating the association between obesity and salivary epigenetic aging in young adult males and females. These findings are of interest to those who are interested in epigenetic age acceleration as a potential biomarker. They also support future research examining obesity as a causal risk factor for epigenetic age acceleration. The findings underscore the importance of testing sex differences and including multiple epigenetic clocks in future research. Overall, the present results add to mounting evidence that obesity affects cellular aging across multiple tissues early in the lifespan.

## METHODS

### Participants and procedures

This study includes 290 young adults (Mean age 27.39, SD = 1.20; 60% female; 80% African American, 18% White, 2% Other) who participated in Wave 4 of the Birmingham Youth Violence Study [35]. The larger study recruited 704 children from 5th grade classrooms in 17 public schools in Birmingham, Alabama, from 2004–2005 and followed them over time. This report only uses data from Wave 4 collected in 2018–2021. Compared to those lost to follow up since Wave 1, the 290 participants retained in Wave 4 were more likely to be female (60% vs. 39%; χ^2^_(1)_ = 30.47, *p* < .001), African American (82% vs. 75%; χ^2^_(1)_ = 4.87, *p* = .027), and with higher parental education (M = 4.30 vs. 4.01, *t*_(691)_ = 2.21, *p* = .027).

After providing written informed consent, participants completed individual interviews in a private interview room at a university lab with trained interviewers utilizing computer-assisted interview procedures. They also provided a saliva sample for epigenetic analyses and had their height, weight, and waist circumference measured. All procedures were approved by the Institutional Review Board and participants were financially compensated for their time.

### DNA extraction and methylation

Saliva samples were collected with Oragene DNA OG-500 kits. DNA was extracted using the PureGene extraction method (Qiagen) following the manufacturer’s specifications. All samples yielded over 2.1 μg of high-quality DNA. Methylation analysis of DNA was performed with the Illumina Infinium MethylationEPIC BeadChip. Normalization and quality control (QC) were conducted in the R package minfi [36] and included probe level QC, sample level QC, background correction, within array-normalization, Type I and II chemistry correction, and batch/plate/chip adjustment. Methylation measurements were quantified as Beta-values, defined as the ratio of methylated fluorescent intensity and overall intensity [37]. The reference-based deconvolution method [38] was utilized to correct for differences in cell composition across samples.

### Measures

#### Body mass index (BMI)

Height was measured using a portable stadiometer and recorded to the nearest 0.1 cm. Weight was measured using a portable electronic scale and recorded to the nearest 0.01 kg. Two measurements were taken and if they differed by > 0.5 cm for height or > 0.2 kg for weight, a third measurement was taken. The average of the two closest values was used and BMI was computed from these average height and weight values.

#### Waist circumference

Participants’ waist circumference was measured using a plastic measurement tape. The measurement tape was placed above the participants’ hipbone with any loose fitting clothes being lifted before taking a measurement. For each participant, two measurements were taken and all measurements were reported in centimeters and one decimal place. If the first two measurements were more than 0.5 cm apart, a third measurement was taken. The closest two measurements were averaged with higher values indicating a larger waist circumference.

#### DNA methylation (DNAm) age

Four epigenetic aging biomarkers were used in this study – DNAm Hannum, Horvath, PhenoAge, and GrimAge [5, 11, 13]. These DNAm age biomarkers have been associated with chronological age and all-cause mortality [13]. The Hannum and Horvath methods are sensitive to chronological changes in DNA methylation, whereas PhenoAge assays CpG sites associated with all-cause mortality [13]. Hannum and Horvath DNAm scores have been validated in a multi-ethnic meta-analysis of 13 populations-based cohorts, which found that higher methylation aging was significantly associated with mortality [39]. PhenoAge was validated in five independent multi-ethnic large-scale samples [13].

Hannum, Horvath, and PhenoAge DNAm age scores were calculated through a linear function using the ENmix function in the minfi package, utilizing published intercepts and regression coefficients for DNAm Age Horvath [5], Hannum [11], and PhenoAge [13], respectively. Horvath’s epigenetic clock is comprised of 353 CpG probes and has been validated across multiple tissues [5]. Hannum’s method is computed from methylation values of 71 CpG sites and has been validated with adult whole blood samples [11]. However, the Horvath method was developed with the 450 K microarray and 17 sites are missing from the 850 k microarray [40]. Similarly, the Hannum epigenetic clock is missing 6 requisite loci [40]. PhenoAge was developed with the 850 k microarray and includes 513 epigenetic loci [13]. Finally, GrimAge was calculated via the DNAm Age calculator (https://dnamage.genetics.ucla.edu/), which is compiled from 1030 CpG probes in blood tissues. All mAge biomarkers were coded with higher values indicating older epigenetic age.

#### Covariates

Sociodemographic covariates included age, sex, race (African American vs. White or other), parental education (reported by a parent at a previous wave), and income-to-needs ratio, calculated by dividing reported annual household income by the poverty threshold for the participant’s household size in the year of data collection [41]. Because tobacco smoking is associated with DNA methylation [42] a sum of current and previous (age 18) smoking indicators (coded 0/1) was included. Finally, proportions of cell types found in the saliva samples were estimated for each sample, including CD8T, CD4T, B cells, Monocytes, and Granular cells.

### Data analyses

The amount of missing data, descriptive statistics, and bivariate correlations among all variables were examined in SPSS. The main analyses involved seven hierarchical regression models predicting PhenoAge, GrimAge, Horvath DNAm age, and Hannum DNAm age from BMI or waist circumference and covariates (age, sex, race, income-to-needs ratio, parental education, smoking, and cell types) in Step 1. Sex differences in the associations of BMI or waist circumference with the DNA methylation age indices were tested with interaction terms in Step 2. Significant interactions were followed-up with simple slope analyses. The regression models were conducted in Mplus 8.1 using Full Information Maximum Likelihood (FIML), which preserves the full sample size (*N* = 290) and minimizes bias when data are missing at random (Cham et al., 2017). Sensitivity analyses were conducted with BMI and waist circumference standardized within each sex to account for sex differences in obesity.
